# An Improved Syringe-Pipe for Hypodermic Injection

**Published:** 1869-08

**Authors:** Thomas Buzzard

**Affiliations:** Assistant-Physician to the National Hospital for the Epileptic and Paralysed


An Improved Syringe-Pipe for Hypodermic Injection.—By Dr. Thomas Buzzard, Assistant-Physician to the National Hospital for the Epileptic and Paralysed.—Those of us who are most favorably disposed towards the hypodermic injection of drugs are yet obliged to allow that our patients not unfrequently object very strongly to the pain occasioned by the introduction of the syringe-tube. And we sometimes find it as hard to convince such persons that they are not being hurt as the shoemaker did to prove to Lord Foppington that his shoe was not too tight. "Your lordship," he said, "may please to feel what you think fit; but that shoe does not hurt you. I think I understand my trade!" A syringe-tube which I have lately had made will, I hope, do something to extend the hypodermic method by the facility and the comparative painlessness of its introduction. The ordinary syringe-tube, it will be remembered, is of gold or silver gilt, and is tubular to the very end, which is sharpened as well as the somewhat soft material allows. Steel tubes of the same form are sometimes employed, but they quickly corrode and get choked. It occurred to me that it was quite unnecessary for the syringe-pipe to be tubular *throughout*, and that a gold tube might with advantage be fitted with a *solid* steel spear-point of the best form for penetrating the skin. At my request, Messrs. Meyer and Meltzer, of Great Portland-street, have attached a triangular steel prism to the ordinary gold tube, and thus constructed an instrument which pierces the skin as easily as the "glover's" needle, which is preferred to all others, by many surgeons, and which the termination of the tube closely resembles. The advantages of incorrodibility in its tubular portion, and sharpness of point are thus combined. The point is wiped by simply passing it between the thumb and finger.—*Lancet, March* 20, 1869, *p* 397.

